# How to Manage Taste Disorders

**DOI:** 10.1007/s40136-022-00428-z

**Published:** 2022-09-21

**Authors:** Julien Wen Hsieh, Dimitrios Daskalou, Sonia Macario, Francois Voruz, Basile Nicolas Landis

**Affiliations:** Rhinology-Olfactology Unit, Department of Otorhinolaryngology Head and Neck Surgery, University Hospital of Geneva, Geneva, Switzerland

**Keywords:** Dysgeusia, Taste, Retronasal, Treatment, Smell, Zinc, Depression

## Abstract

**Purpose of the Review:**

This study aims to summarize the current state of the art of how taste disorders are clinically best managed.

**Recent Findings:**

Taste disorders are distressing for the concerned patients since eating and drinking become bothersome or impossible. Apart from nutritional problems, quality of life is impaired. Still, diagnosis and treatment of taste disorders are elusive, and general knowledge about taste and its affection is little within the population and the medical community. This review stresses the importance of accurate workup and diagnosis of taste disorders in order to offer an effective treatment. Yet unclear aspects of taste disorders are discussed, and interesting findings regarding the treatment of taste disorders are reviewed. A special focus is given to current pharmacological options on how to treat taste disorders.

**Summary:**

Despite impressive insights into the gustatory function and molecular logic of taste receptor cells, there is currently poor clinical knowledge on the pathophysiology of taste disorders in humans. Diagnosing, measuring, and treating gustatory disorders remain restricted to a handful of specialized smell and taste centers worldwide. Despite interesting work on potential drugs treating taste disorders, many of the reported medications lack controlled and randomized trials confirming their efficacy in taste dysfunction. Future efforts need to be focused on the treatment of taste disorders.

## Introduction

Taste or gustation is one of the three chemical senses which allow humans to detect their molecular environment [1]. Gustation comprises the basic taste qualities including sweet, sour, salty, bitter, and umami (also known as monosodium glutamate). Further, water has been shown to be processed by taste nerves suggesting it to be the sixth basic taste [2]. Besides gustation, olfaction, and somatosensory afferents (intranasal and intraoral trigeminal nerve) contribute to the decoding of chemical information. The main chemosensory organs are the nose and the oral cavity. Both are innervated by trigeminal fibers providing us with information about texture, temperature, irritation, and concentration of volatile or liquid chemical compounds. Olfactory perception is restricted to the nasal cavity as taste is so to the oral cavity. Thus, the nose (olfaction and trigeminal) and the oral cavity (taste and trigeminal) are both double sensory organs, which is a circumstance that has to be considered when these senses are tested. In most daily life situations (e.g., inhaling surrounding air, eating, or drinking), a simultaneous stimulation of two or all three modalities (smell, taste, trigeminal) takes place. People thus often mix up trigeminal, olfactory, and gustatory stimulations [3], and these senses interact mutually in health [4] and disease [5•]. A further confusing element in the perception of chemosensory information is that odorous molecules can reach the olfactory epithelium not only via the nostrils but also via the nasopharynx through the so-called retronasal way. Orally applied substances (e.g., cinnamon in a candy) are present in the oral vapor phase, and this odorized air is projected into the nasal cavity when we swallow [6, 7]. At that moment, we perceive the flavor of cinnamon. Since this odorous information is experienced through an oral stimulation, most people mistake this for a “taste” percept. This misinterpretation influenced the verbal use of taste and smell experiences. Most flavor perceptions are called a “taste” regardless of how much real sweet, salt, sour, bitter, or umami components influence this percept. Patients complaining of smell, taste, or flavor disorders should be investigated by individual testing of each of the three modalities to avoid misinterpretation and misdiagnosed of taste and smell disorders [3, 8]. For smell, taste, and intranasal trigeminal sensitivity, quick and easy-to-perform psychophysical tests are available [1, 9••]. In contrast, intraoral trigeminal testing is neither yet standardized nor routinely performed.

## Anatomy and Physiology

The discovery and characterization of these receptors have largely been achieved during the last two decades [2, 10]. Besides the aforementioned basic tastes and water, it is still debated whether fat recognition is a taste stimulus or rather a somatosensory stimulus [11].

**Fig. 1:**
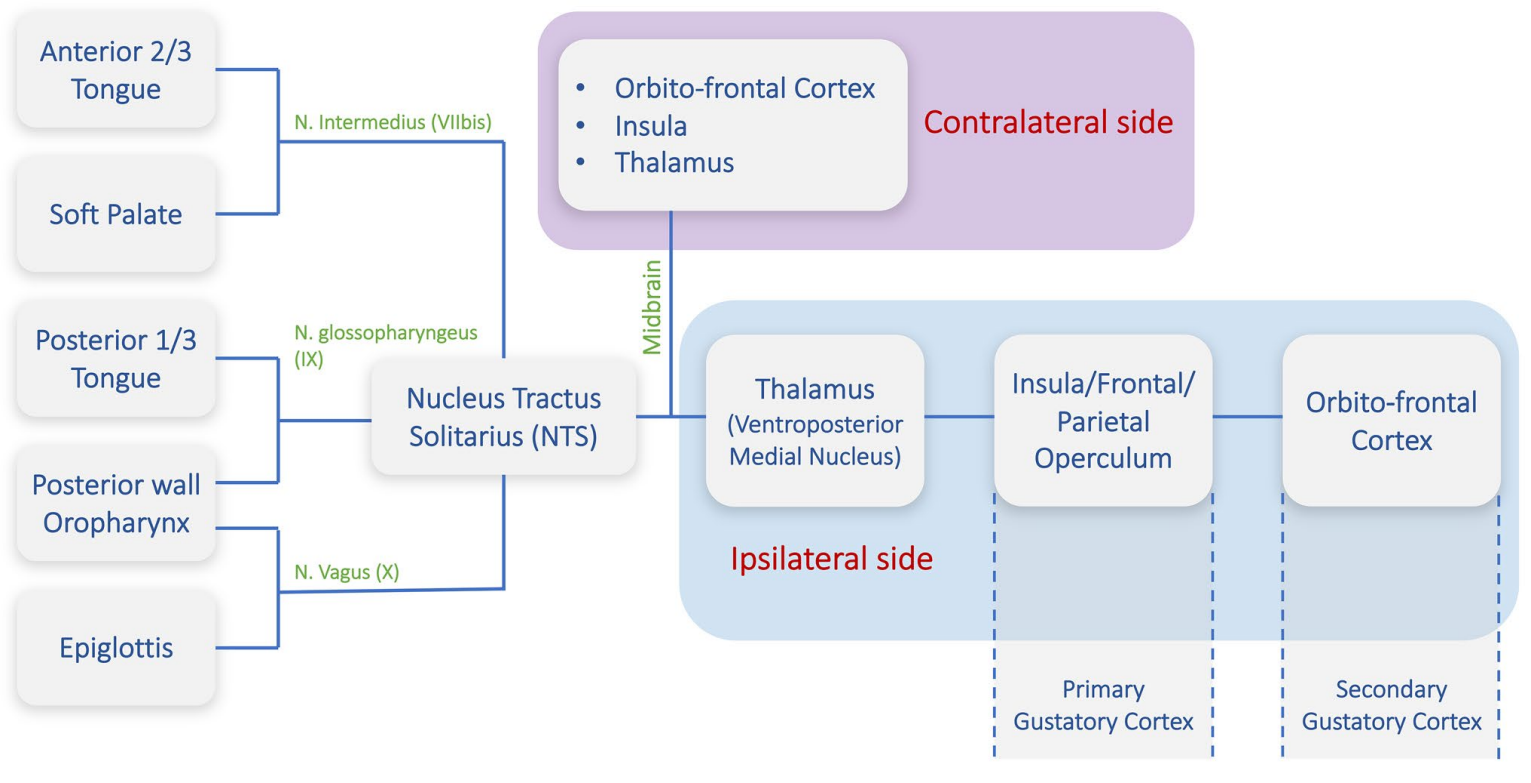
Overview of the currently accepted human taste pathway

The taste receptors are located on the tongue, the soft palate, the epiglottis, and the rear of the pharynx and the larynx. The three cranial nerves VIIbis (intermediate nerve, part of the facial nerve also called nerve of Wrisberg or VIIbis), IX (glossopharyngeal), and X (vagus) contain the gustatory fibers for the mentioned regions. Their gustatory fibers converge at the brain stem level into the nucleus tractus solitarius (NTS) (Fig. 1). Then, taste pathway projects bilaterally to the thalamus [12, 13] and to the primary (insula, frontal, and parietal operculum) and later to the secondary gustatory cortex (orbitofrontal cortex) (Fig. 1).

## Taste Disorders and Their Management

The challenges of managing taste disorders are (a) correct identification of a taste (and not smell/retronasal) disorder, (b) properly measuring taste function, (c) establishing a diagnosis, and (d) offering a treatment option.

Only a handful of epidemiological studies have been published on the frequency and putative causes of taste disorders, and many patients remain with the rather vague diagnosis of idiopathic taste disorder [14–20]. This reflects, in contrast to olfactory disorders, the still moderate clinical knowledge we have about taste disorders and the low prevalence of routine taste testing in clinical settings.

### Clinical Presentation and Classification of Taste Disorders

**Fig. 2:**
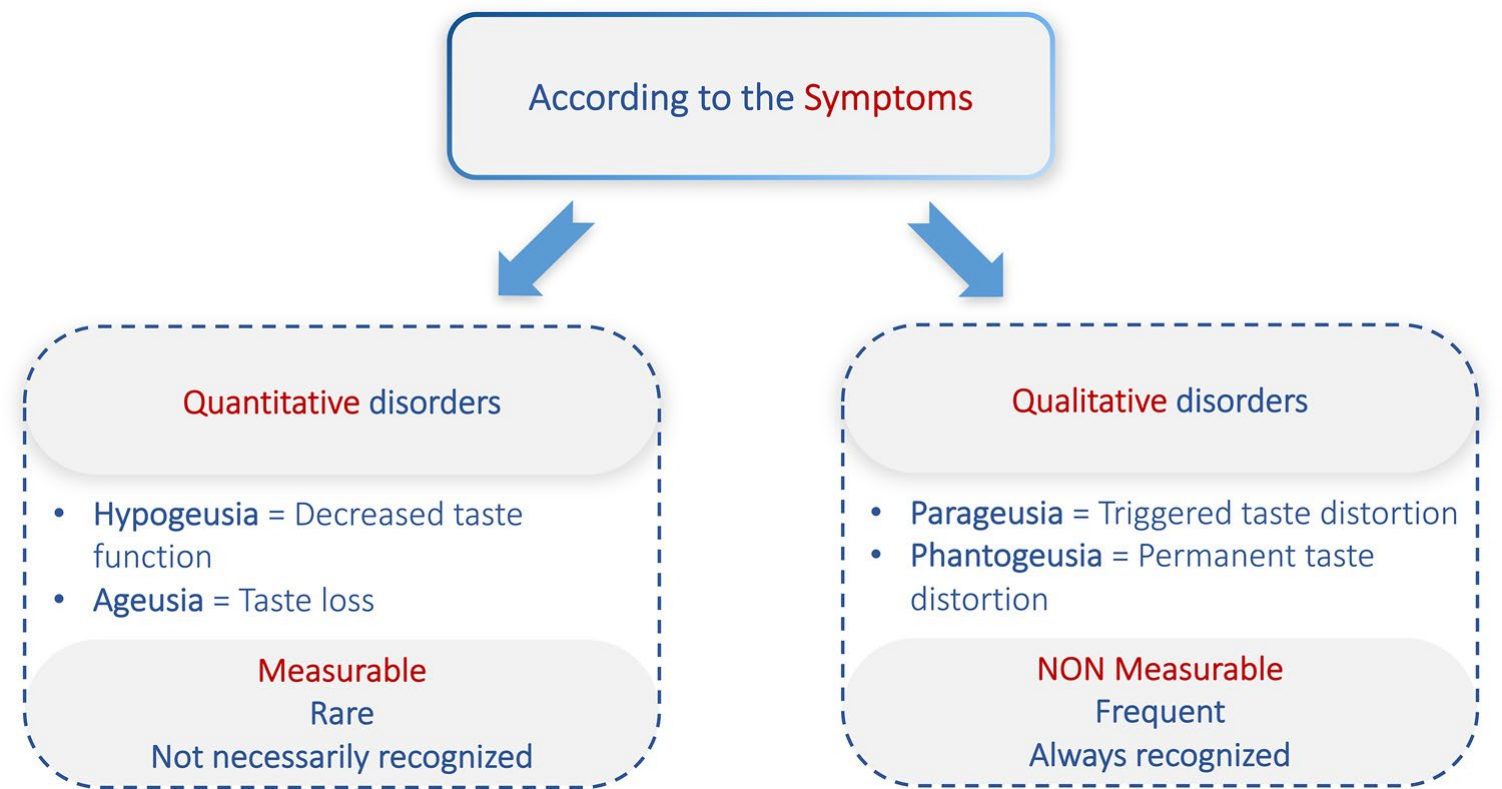
Classification of taste disorders based on the clinical presentation of the symptoms

Based on patient’s complaints, taste disorders can be divided into qualitative versus quantitative disorders (Fig. 2). Qualitative taste disorders are most often noticed by patients since they affect the daily life by permanent or triggered distortions of taste sensations. In contrast, quantitative taste disorders do not necessarily produce strong complaints. Remarkably, decreased taste function seems to be more prevalent in the population than previously thought, without being noticed by the concerned people [14], and thus, measuring gustatory function is of paramount importance. Many patients do not only present with qualitative or quantitative affection but also have mixed complaints. The word dysgeusia, which should describe all qualitative and quantitative taste disorders as a general term, is used very variably amongst authors and is most frequently taken to report qualitative, distorted taste perception.

**Fig. 3:**
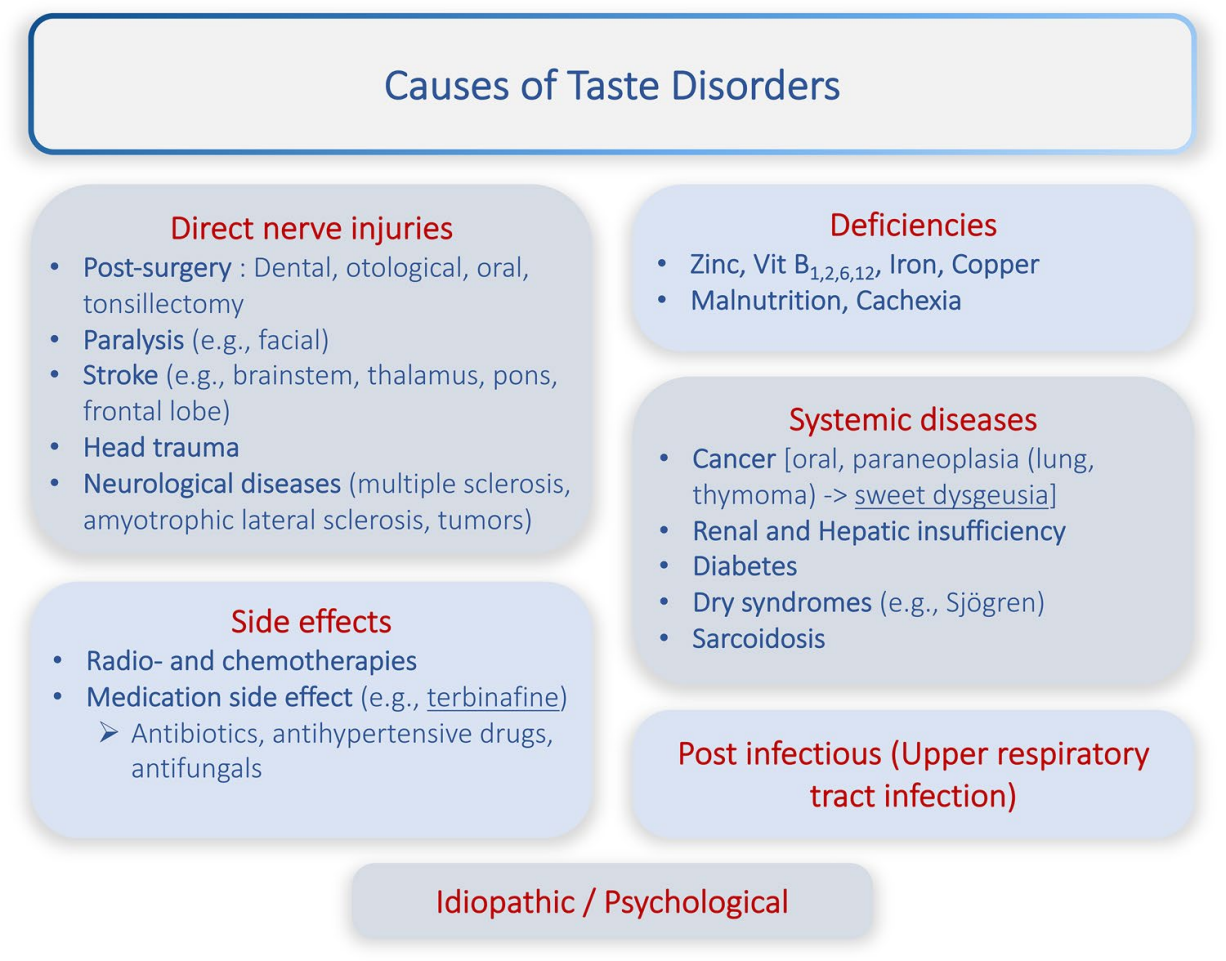
Overview of the current most frequently encountered differential diagnosis for taste disorders

Furthermore, according to the classification based on the clinical presentation, taste disorders might be classified according to the *site of lesion* or to the *most likely cause*. The anatomical site of the lesion determines whether it is a central nervous or a peripheral taste disorder. Lesions distal to the NTS (Fig. 1) are considered peripheral, whereas NTS and more proximal lesions are central nervous lesions. Currently, the most used classification in clinical routine is to diagnose taste disorders according to their most likely cause (see Fig. 3). There are roughly 5–6 major etiological groups (neural injuries, deficiency states, medication side effects, postinfectious, systemic diseases, and idiopathic) which have to be inquired during the patients history. Since the treatment of a taste disorder mainly depends of its cause, the correct diagnosis is the key to a successful treatment. Different kinds of neural injuries ranging from peripheral to central and from trauma, inflammation, irradiation, stroke, to surgical injury have been described to cause qualitative and/or quantitative taste disorders [21, 22]. The gustatory system seems to be particularly vulnerable to metabolic and systemic disease states. As a consequence, taste disorders frequently occur after medication intake, deficiency states, or as an epiphenomenon of systemic diseases [23]. Angiotensin-converting enzyme inhibitors, one of the most commonly prescribed drugs for the management of hypertension and congestive cardiac failure, cause qualitative taste disorders by increasing the local bradykinin concentration and by inducing deficiency of zinc and copper. Other widely used medications that affect taste are the diuretics, like amiloride, spironolactone, furosemide, and lipid-lowering agents like atorvastatin and simvastatin [24–26]. Rarer causes for dysgeusia are food components (e.g., pine nuts [27, 28]) or paraneoplastic symptoms. Especially sweet dysgeusia should be investigated very cautiously by employing a chest scanner as it has repeatedly been described as the first symptom of lung or thymus cancer [29, 30]. The remaining large group of patients is still diagnosed with idiopathic taste disorders, which basically means that after having ruled out all possible other causes, no clear origin could be found. This highlights the moderate clinical knowledge and the future challenges in clinical taste disorders we have to overcome.

### Burning Mouth Syndrome (BMS)

A special form of taste disorders is the so-called burning mouth syndrome. It is characterized by burning, dry, and often painful oral discomfort. Gustatory complaints are not mandatory and only accompany 30% of the BMS cases [31]. This condition is more frequent in postmenopausal women. Its exact origin is still unclear, and psychological factors aggravating the situation are suspected [32]. Recent neurophysiological, psychophysical, pathological, and functional MRI studies suggest several neuropathological mechanisms involved in primary BMS [33]. Experimental data support different hypotheses going from BMS being a peripheral neuropathy, central neuropathy, or even a phantom pain symptom [32]. The loss of small diameter nerve fibers in the oral cavity is one element yet identified to be a key element in the pathogenesis of BMS. However, it is not clear what causes this nerve fiber loss [33]. BMS is consequently not an exclusive taste pathology but rather affects taste perception by the interaction between the concerned sensitive, trigeminal closely located taste fibers. Its treatment is also more similar to that of chronic pain disorders [34].

### Measurement of Taste Disorders

The measurement of taste function is a key element in the workup of taste disorders. As previously mentioned, qualitative taste complaints cannot be measured (Fig. 2). Like any sensory modality, the gustatory function is assessed either objectively or psychophysically. Objective measurement tools are functional MRI [35], PET scans, and gustatory event-related potentials [36, 37]. These tools are either restricted to research settings or time-consuming which makes them unsuitable for routine clinical practice. Gustatory event-related potentials are a helpful tool for medico-legal issues in order to assess objectively taste function [36, 38]. They are currently used only for particularly difficult clinical cases, but efforts are underway to make them more user-friendly for clinical routine.

Psychophysical testing of gustatory function has been more extensively studied. In the past, taste tests were self-made and individually done by each clinic, which basically meant that everybody had a different gustatory testing device. In the last two decades, there have been efforts done by the German Smell and Taste Working group to standardize these procedures. Consequently, the taste strips have been developed [39] and validated [40]. They consist of spoon-shaped filter papers which are impregnated with salt, sugar, quinine, or citric acid. This test allows for multicenter studies and can easily be transported or sent by post. Further advantages are the possibility to test each side of the tongue separately and thus unravel lateralized taste deficit, which would go unnoticed with whole mouth testing [41]. Alternatively, taste sensations can be elicited with electric stimulation to the tongue. This principle has been used with the electrogustometry [42], which is easy and quick but has the disadvantage of co-stimulating lingual trigeminal somatosensory fibers leading to a mixed taste trigeminal perception [43].

Testing gustatory function is influenced by verbal biases. In contrast to odor identification tasks, normal and healthy subjects have been repeatedly shown to exhibit many difficulties with correctly identifying basic tastants. Sugar is identified as being sweet by over 95% of the patients, but when it comes to salty, sour, and bitter, these three tastes are constantly mistaken for another one [14, 44].

### Clinical Exam/Imaging/Labwork

To ascertain the diagnosis of the taste disorder is crucial for the choice of a treatment. As mentioned above, the patient’s history (see Fig. 3) should look for elements explaining the taste disorder. Besides formal testing of smell, taste, and if possible trigeminal nerves, a thorough clinical ear, nose, and throat and neurological examination is mandatory. Imaging can be indicated in cases of suspicion of central nervous or otherwise idiopathic taste disorders. The most suitable imaging would be an MRI in order to visualize the gustatory pathway. In case of normal examination and imaging, we emphasize the routine blood sample looking for any major metabolic dysregulation or deficiency (e.g., iron, thyroid, renal or hepatic dysfunction, and vitamins) states or underlying immunological diseases (e.g., Sjögren, sarcoidosis, inflammation) [45].

## Treatment

Before giving an overview of the so far published and clinical useful knowledge on treatment of the taste disorders, it has to be stated that (a) the treatment is always dependent on the cause of the taste disorder and that (b) taste disorders have been neglected for a long time by the medical community with the consequence of yet very few possible and well-investigated treatments.

### Non-pharmacological Treatment Options

#### Spontaneous Recovery

Unlike other sensory systems (e.g., vision or audition), the gustatory system has a high degree of spontaneous recovery due to the normal regeneration rate in health and disease [46, 47]. The recovery rates are high, but the time range for full recovery is up to 2 years. This information has to be given to the patient, and follow-up is required to ascertain the recovery. For almost all cases of *nerve injury* or *postinfectious taste impairment (also COVID-19-induced)*, the first-line treatment is to inform the patient and to wait for the spontaneous recovery to take place [48]. If this should not be the case, pharmacological treatments could be tried.

#### Medication Discontinuation

In cases of taste disorders due to *medication or food side effects*, the only meaningful therapy is the discontinuation of the medication. Despite discontinuation, it might take several days and sometimes up to months until the taste function returns to normal again. A very prevalent example is terbinafine, an oral antifungal drug, which causes taste disorders classically 3–5 weeks after it is started. Once stopped, it takes another 5–6 weeks until taste function normalizes [49].

#### Transcranial Magnetic Stimulation

Repetitive transcranial magnetic stimulation, used to treat depression, has been reported successful in some cases of smell and taste disorders [50]. However, this treatment has not yet found its way into the routine clinical management of taste disorders.

### Evidence Level of Pharmacological Treatments/Quality of Trials

In contrast to other areas of medicine, little knowledge and few studies are reported on treatment options for taste disorders. As mentioned above, the main efforts of clinicians dealing with taste disorders are currently directed to diagnosis and identification of the underlying cause. Consequently, few authors have so far conducted prospective studies with a pharmacological treatment for *idiopathic* gustatory dysfunction. Besides some anecdotal or small sample-sized and uncontrolled successful treatments with ice cube suction [51], local anesthesia [52], theophylline [53], alpha-lipoic acid [54], or vitamin D substitution [55], few drugs have been identified to be reliable and valuable treatment options for taste disorders. The literature shows that basically two groups of medications revealed repeatedly helpful in the treatment of taste disorders, namely, zinc and antidepressants.

#### Antidepressants

Several publications suggested that there is a link between mood state and taste complaints. Deems showed that spontaneous recovery rates for idiopathic dysgeusia were significantly better in patients with positive mood states compared to those close to depression [56]. Bonfils et al. reported positive effects of cognitive behavioral therapy on glossodynia and dysgeusia [57], and Amsterdam et al. directly showed a correlation between depression and taste function [58]. Treatment of depression seems thus to have an effect on taste disorders, confirming the view that *idiopathic* dysgeusia might be a neglected symptom of mild depression [59]. Interestingly, human taste function responds to antidepressants not only in disease but also in health. Heath and collaborators investigated the effect of several selective serotonin re-uptake inhibitors and noradrenaline on human taste thresholds and found significant ameliorations after administration of all antidepressants [60]. The study was placebo-controlled with no effect of the placebo. This raises the question to which extent taste function could generally be improved by antidepressants and if such a treatment could be used for dysgeusia patients unrelated to depression. However, it remains unclear if taste disorders may cause depression or vice versa depression is sometimes accompanied by taste disorders. As stated above, certain forms of oral disorders such as BMS seem to respond to neuro-modulators such as clonazepam [34]. For pure taste disorders, not related to BMS, only anecdotic reports for amitriptyline [61], valproate [62], and gabapentin [63] seem to suggest that these drugs may be used successfully. These cases just underline the need for bigger and more evidence based therapeutic approaches in pharmacologic treatment of taste disorders [64].

#### Zinc Gluconate

Zinc is probably the only well-investigated drug proposed for the treatment of *idiopathic* taste disorders. Although zinc has been successfully given to patients with postoperative taste disorders, this indication remains based on case reports [65]. In contrast, the application of zinc gluconate for *idiopathic taste disorders* is very well documented with several double-blind randomized clinical trials clearly showing that intake of zinc gluconate significantly improved taste disorders. The first double-blind randomized trial was conducted by Yoshida et al. [66], followed by Heckmann et al. [67] who both showed a clear improvement in taste disorders after 3 months of zinc gluconate oral intake (140 mg/daily). Since the side effects are relatively harmless (nausea and gastrointestinal problems) and occur only in doses far above the reported daily regimens, zinc gluconate therapy offers a good treatment option for idiopathic taste disorders. The zinc effect has further been supported by the work of Sakagami et al. who used a zinc-containing molecule polaprezinc [68] instead of zinc gluconate. The problem with zinc prescription in patients having taste disorders is the lack of correlation between serum and saliva zinc levels, symptoms, and response to treatment. Some patients complain of taste disorders but have normal zinc serum levels, whereas deficient patients do not have any complaints. Further, the zinc status before treatment is not a reliable prognostic factor for treatment success [67]. Having said this, it underlines the current open question of why zinc works with taste disorders. Besides some hypotheses that zinc might serve as a crucial co-factor for saliva proteins which in turn influence the growth and turnover of taste cells [69], it has been hypothesized that antidepressant effects of zinc could account for the observed effects [70].

Zinc has been shown to enhance the effect of antidepressant drugs [71], and it is speculated that zinc alone could also have weak antidepressant properties [70]. By having a close look at the before-mentioned clinical trial data done by Heckmann et al. [67], it confirms this idea. The zinc-treated group improved their Beck Depression Inventory significantly compared to the placebo group. As seen and suggested in the previous paragraph, antidepressant properties seem to influence the taste function positively in health and disease, thus explaining the zinc effects. However, the same zinc doses proven to be efficient in dysgeusia patients did not have any preventive and protective effect in a double-blind trial on chemotherapy-induced taste disorders [72], indicating the restricted indication of zinc treatment only for idiopathic cases. Another interesting finding regarding the importance of zinc in taste pathophysiology is provided by Fukasawa et al. [73], who went through the literature on medication side effects. He correlated the number of reported side effects of a given drug with its zinc-chelating properties and found a significant association, which suggests that the more a drug chelates zinc, the more it is likely to see taste side effects from this drug.

## Conclusions

Taste disorders can be very cumbersome for the concerned patients. In contrast to other sensory impairments such as visual or hearing problems, little knowledge is present amongst the general population as well as in most health professionals. Consequently, most patients take much efforts and often quite some time to be referred to a specialized smell and taste center. Such a referral is important for the patient to be taken seriously and counseled professionally, as the treatment depends upon an adequate diagnosis. Unfortunately, the current knowledge about the differential diagnosis of taste disorders remains small, and considerable efforts will have to be made in future research to improve diagnostic accuracy and therapeutically success of taste disorders.
